# Tau Protein-Induced Microtubule Degeneration and Quantum Coherence Disruption: Implications for Memory, Consciousness, and Temporal Integration in Alzheimer’s Disease

**DOI:** 10.1192/j.eurpsy.2026.11260

**Published:** 2026-07-31

**Authors:** H. Yildiz

**Affiliations:** Turkish Psychiatric Association, ankara, Türkiye

## Abstract

**Introduction:**

Tau protein hyperphosphorylation and aggregation in Alzheimer’s disease (AD) lead to microtubule destabilization, impairing neuronal transport and structural integrity. Emerging theoretical models suggest that microtubules may also support quantum coherence states, potentially contributing to higher-order cognitive processes such as temporal binding and unified consciousness.

**Objectives:**

We propose a **Microtubule–Consciousness Gate Model** in which neuronal microtubules serve not only as cytoskeletal scaffolds but also as *quantum-temporal conductors.* At birth, these structures are densely covered by receptor-like nanostructures—termed **Consciousness Gates**—that regulate the flow of coherent information across the brain’s quantum–classical interface.

**Methods:**

**1. Study Design**

This research adopts a **multimodal approach** integrating neuroimaging, molecular assays, electrophysiology, and computational modeling. The primary aim is to evaluate the structural integrity, functional dynamics, and quantum coherence potential of neuronal microtubules under healthy and pathological conditions.

**Results:**

The present work proposes a novel integrative framework in which **neuronal microtubules** function not only as structural and transport elements but also as **temporal–cognitive–mnemonic conduits**. This model emphasizes the existence of **receptor-like gating structures** densely distributed along the microtubule lattice at birth, potentially numbering in the billions. These gates may operate as **binary regulators** of consciousness-related information flow, analogous to semiconductor switches in quantum computing architectures.

Our theoretical formulation posits that these gates are **susceptible to both reversible and irreversible modulation** by environmental, linguistic, and biological factors.

**Conclusions:**

This study introduces a novel framework positioning **neuronal microtubules** as dynamic conduits of temporal, cognitive, and mnemonic flow, regulated by gate-like receptor structures that are highly sensitive to linguistic, biological, and environmental modulation. The theoretical model offers a molecular-to-network explanation for how disruptions in these gates could underlie temporal disorientation, memory impairment, and altered consciousness. By integrating concepts from **molecular neuroscience, quantum biology, and cognitive science**, this hypothesis bridges micro-scale structural changes with macro-scale cognitive phenomena.

The implications are twofold: (1) **biomarker potential**, wherein early detection of gate dysfunction could predict neurodegenerative progression; and (2) **therapeutic innovation**, where pharmacological, cognitive-linguistic, and electromagnetic strategies could restore gate function. Future empirical validation of this model could reshape our understanding of consciousness-related disorders and open new avenues for targeted intervention.

**Disclosure of Interest:**

None Declared

